# Upregulating ANKHD1 in PS19 Mice Reduces Tau Phosphorylation and Mitigates Tau Toxicity-Induced Cognitive Deficits

**DOI:** 10.3390/ijms26157524

**Published:** 2025-08-04

**Authors:** Xiaolin Tian, Nathan Le, Yuhai Zhao, Dina Alawamleh, Andrew Schwartz, Lauren Meyer, Elizabeth Helm, Chunlai Wu

**Affiliations:** 1 Neuroscience Center of Excellence, Department of Cell Biology and Anatomy, Louisiana State University Health Sciences Center, New Orleans, LA 70112, USA; 2 Louisiana State University Health Sciences Center, School of Medicine, New Orleans, LA 70112, USA; 3 LSU Health Shreveport, School of Medicine, 1501 Kings Highway, Shreveport, LA 71103, USA

**Keywords:** ANKHD1, Tau phosphorylation, PS19 mouse, cognitive deficits

## Abstract

Using the fly eye as a model system, we previously demonstrated that upregulation of the fly gene *mask* protects against FUS- and Tau-induced photoreceptor degeneration. Building upon this finding, we investigated whether the protective role of *mask* is conserved in mammals. To this end, we generated a transgenic mouse line carrying Cre-inducible *ANKHD1*, the human homolog of *mask.* Utilizing the TauP301S-PS19 mouse model for Tau-related dementia, we found that expressing ANKHD1 driven by CamK2a-Cre reduced hyperphosphorylated human Tau in 6-month-old mice. Additionally, ANKHD1 expression was associated with a trend toward reduced gliosis and preservation of the presynaptic marker Synaptophysin, suggesting a protective role of ANKHD1 against TauP301S-linked neuropathology. At 9 months of age, novel object recognition (NOR) testing revealed cognitive impairment in female, but not male, PS19 mice. Notably, co-expression of ANKHD1 restored cognitive performance in the affected female mice. Together, this study highlights the novel effect of ANKHD1 in counteracting the adverse effects induced by the mutant human Tau protein. This finding underscores ANKHD1’s potential as a unique therapeutic target for tauopathies.

## 1. Introduction

The microtubule-associated protein Tau is a key component of fibrillary aggregates found in neurons and glial cells of patients with a range of neurodegenerative disorders collectively known as tauopathies [1,2]. Under normal physiological conditions, Tau functions as a microtubule-binding protein that regulates microtubule dynamics, with over 80% Tau bound to microtubule (MT) [3]. Through interactions with MTs and motor proteins such as Kinesin and Dynein, neuronal Tau plays an important role in stabilizing MTs and facilitating axonal transport of cargo from the soma to the presynaptic terminal [4]. However, in tauopathies, Tau undergoes abnormal post-translational modifications, leading to the formation of fibrillary aggregates in both neurons and glial cells. These aggregates, known as paired helical filaments (PHF), are twisted, rope-like structures composed of misfolded Tau proteins [2,5]. The accumulation of PHF-Tau is a hallmark of tauopathies—including Alzheimer’s disease, frontotemporal dementia, and corticobasal degeneration—and is considered a major contributor to the disease pathology. Importantly, these toxic PHF-Tau aggregates can propagate in a prion-like manner, spreading within cells and across cell boundaries, as demonstrated in both cell culture studies [6,7] and mouse models [8]. Emerging research indicates that early Tau abnormality—such as mislocalization, oligomerization, and change in solubility—are more strongly associated with neurotoxicity than the formation of mature fibrils [2,9,10,11]. As such, strategies aimed at enhancing Tau clearance during the early, pre-symptomatic stages of disease represent a promising avenue for therapeutic intervention.

Autophagy plays a vital role in clearing aggregated proteins and serves as a primary mechanism for maintaining neuronal health. Impairment of the autophagy-lysosomal pathway has been identified in the brains of patients with tauopathies [12,13,14] and in various animal tauopathy models [15]. In mouse models, pharmacological activation of autophagy has been shown to reduce the levels of accumulation and spread of misfolded proteins, thereby mitigating associated neuronal loss, underscoring the therapeutic potential of autophagy modulators [15,16,17]. Our recent study in *Drosophila* demonstrated that Mask is both necessary and sufficient for promoting the autophagy-lysosomal pathway [18]. In a fly model of tauopathy, upregulation of Mask in photoreceptor neurons suppressed, while downregulation of Mask enhanced, the eye degeneration induced by expressing human mutant Tau in the photoreceptors [18]. These findings indicated that Mask-mediated enhancement of the autophagy pathway plays a cytoprotective role against degeneration induced by toxic protein aggregates in flies. However, it remains unclear whether this protective mechanism is conserved in higher organisms. Specifically, it is unknown whether the mammalian homolog of Mask can influence the progression of neurodegeneration.

The human genomes encode two *mask* homologues, *ANKRD17* and *ANKHD1*. Loss of function of *ANKRD17* has been linked to neural developmental disorders [19,20,21], while ANKHD1 is frequently upregulated in a variety of tumors [22,23,24,25,26,27]. Studies into the role of ANKHD1 in cancer have identified it as a key driver of uncontrolled cellular proliferation, enhanced tumorigenicity, accelerated cell cycle progression, and increased epithelial-to-mesenchymal transition, all of which contribute to enhanced tumor thriving [23,26,28,29,30,31]. Structurally, ANKHD1 contains 25 ankyrin repeat motifs divided into two distinct domains, as well as a single KH domain. When aligned with Mask, the first ankyrin domain set shares 79% sequence similarity, the second set 63%, and the KH domain 54%, indicating a high degree of conservation. Given ANKHD1’s pro-survival function in cancer cells and its high sequence homology with Mask, we hypothesize that ANKHD1 exerts a conserved neuroprotective role like its *Drosophila* homolog *mask*. Our data show that neuronal expression of ANKHD1 in transgenic mice expressing the human mutant P301S Tau attenuated hyper-phosphorylated Tau accumulation, moderately mitigated the loss of presynaptic markers in the hippocampus, and partially restored the cognitive deficits caused by the mutant Tau protein.

## 2. Results

### 2.1. Generation and Validation of Cre-Inducible ANKHD1 Expression Mouse Lines

**Figure 1:**
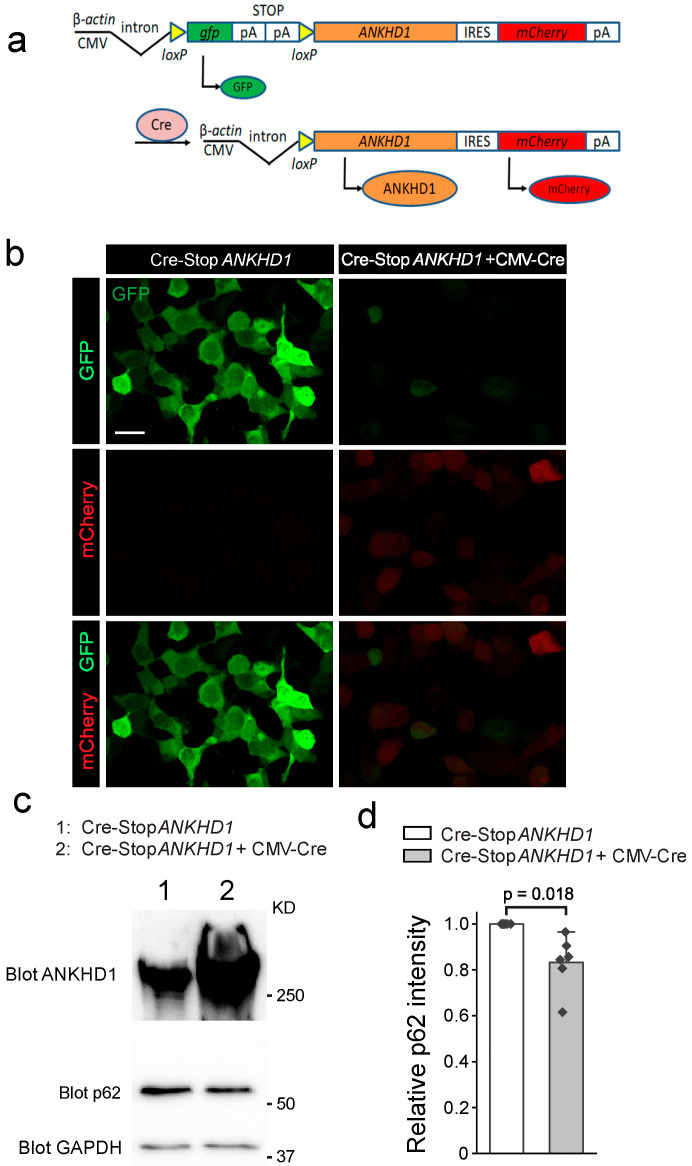
Generating Cre-inducible *ANKHD1* transgenic mice. (**a**) Schematics of the DNA construct for generating Cre-inducible *ANKHD1* transgenic mouse. (**b**) Representative confocal images of GFP and mCherry auto-fluorescence from HEK293 cells transfect with Cre-Stop-*ANKHD1* with (right) or without (left) CMV-Cre plasmid. Scale bar, 20 μm. (**c**) Western blots of cell lysates from the two groups of HEK293 cells showing in **b** with anti-ANKHD1, p62, and GAPDH antibodies (as loading control). (**d**) Quantification of p62 intensity normalized to GAPDH (n = 6).

To generate a Cre-inducible *ANKHD1* transgenic line that can be used to aid the test of its effects in mitigating neurodegeneration, we first designed and constructed the Cre-inducible *ANKHD1* expression construct under the CMV promoter as depicted in Figure 1a. The design of this construct allows the cell-type-specifically expressed Cre recombinase to remove the *loxp*-flanked GFP STOP cassette, and therefore, permits the expression of the human ANKHD1 as well as the mCherry protein whose coding sequence follows *ANKHD1* and an IRES signal (Figure 1a). Before this Cre-inducible *ANKHD1* construct was used to generate transgenic mouse colonies, we transfected HEK293 cells with the construct along with a CMV-Cre plasmid. As anticipated, in the absence of CMV-Cre, most of the cells strongly expressed GFP proteins (Figure 1b left). In the presence of CMV-Cre, GFP expression was largely diminished, and modest mCherry expression was detected in the cells (Figure 1b right). We predicted that mCherry-expressing cells also express ANKHD1, which was confirmed by Western blot using anti-ANKHD1 antibody (Figure 1c).

To assess the impact of ANKHD1 on autophagy flux, we measured the levels of p62, a well-established marker of autophagy activity. Under normal conditions, p62 facilitates the transport of damaged cellular components to autophagosomes and is subsequently degraded in the autolysosomes. We observed significant reduction in p62 protein levels in ANKHD1 expressing HEK293 cells (Figure 1c,d), suggesting that ANKHD1 overexpression elevates autophagy flux. This finding aligns with our hypothesis that ANKHD1, like its fly homolog Mask, can promote autophagy.

**Figure A1:**
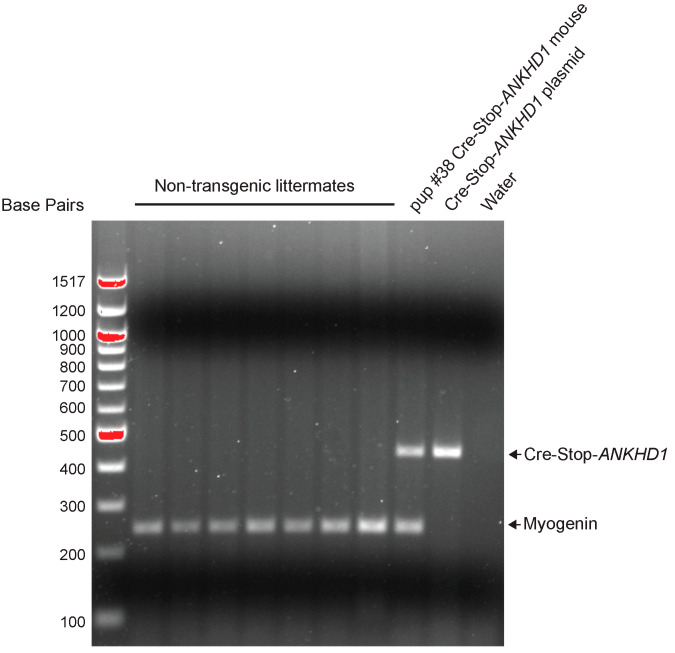
Genotyping of the Cre-Stop-*ANKHD1* mouse. Genomic DNAs from the punched ear tissues of transgenic and non-transgenic pups were extracted and subjected to PCR genotyping with primer pairs recognizing *Myogenin* and the transgenic *Cre-Stop-ANKHD1.* Cre-Stop-ANKHD1 DNA plasmid was used as positive control and water was used as a negative control.

The Cre-inducible *ANKHD1* constructs were then used to generate transgenic mice. We obtained three founder lines, #38, #48, and #62. Founder line #38 was chosen for the rest of this study due to our early identification and establishment of this colony (Figure A1).

To test whether ANKHD1 is capable of conferring a conserved protective effect in neurons, we crossed the Cre-inducible *ANKHD1* transgene into the previously established Tau^PS19^ (PS19), or pnp-Tau-P301S mouse model [32], and generated a cohort for further examination on the neuropathological hallmarks in the mouse brains and mouse behaviors that reflect their cognitive functions. We generated this cohort of mice littermates that contains the control mice (+/+; CamK2a-Cre/+), the Tau^PS19^ mice (pnp-Tau-P301S/+; CamK2a-Cre/+), and Tau^PS19^+*ANKHD1* mice (Cre-Stop-*ANKHD1*/+; pnp-Tau-P301S/+; CamK2a-Cre/+) through two sequential crosses: the first cross uses the Tau^PS19^ and the CamK2a-Cre as the parental mice; and the second cross uses the resulting pnp-Tau-P301S/+; CamK2a-Cre/+ double heterozygous progenies from the first cross to mate with the Cre-Stop-*ANKHD1* transgenic mice.

**Figure 2:**
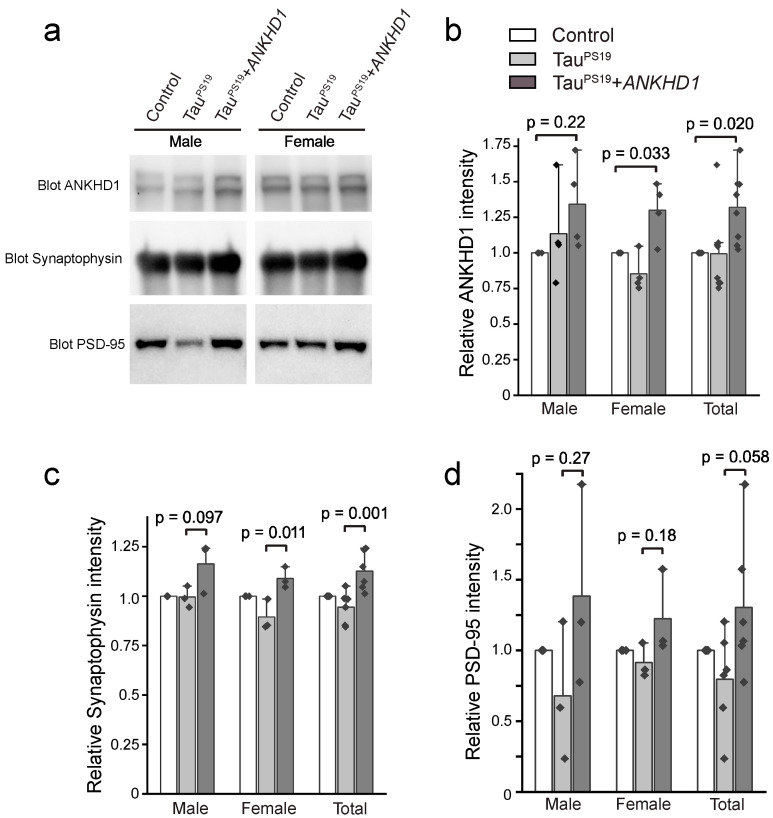
ANKHD1, Synaptophysin, and PSD-95 abundance in the brains of control, TauP301S, and TauP301S + *ANKHD1* mice at 6 months of age. (**a**) Representative Western blot images of brain hippocampal lysate from the Control, Tau^PS19^, and Tau^PS19^+*ANKHD1* mice. Anti-ANKHD1, anti-Synaptophysin and anti-PSD95 antibodies were used to detect and measure the levels of ANKHD1, and the pre- and post-synaptic markers. Quantification of (**b**) ANKHD1 (n = 4), (**c**) Synaptophysin (n = 3), and (**d**) PSD95 (n = 3) intensities on the immunoblot. The same amount of total protein was loaded to each lane.

We first confirmed the ANKHD1 expression in the hippocampus region in 6-month-old mouse brains. We found that the ANKHD1 levels show a moderate 40% increase in hippocampus (N = 4, *p* = 0.02 for Tau^PS19^+*ANKHD1* vs. control mice, and *p* = 0.017 for Tau^PS19^+*ANKHD1* vs. Tau^PS19^ mice). However, when the male and female mice were analyzed separately, although they show the same trend of increased ANKHD1 levels, only female mice showed statistically significant differences (Figure 2a,b).

### 2.2. ANKHD1 Suppresses Neuron-Specific Histopathological Lesions Induced by Mutant Human Tau Proteins in the Mouse Brain

Loss of synapses in the hippocampal region in the Tau^PS19^ mice were previously reported [33,34], we then examined the levels of the presynaptic marker Synaptophysin and the postsynaptic marker PSD95 in the hippocampal lysate from 6-month-old mouse brains. We found that, when compared to the Tau^PS19^ mice, the Tau^PS19^+*ANKHD1* mice moderately restored the levels of Synaptophysin (N = 3, and *p* = 0.001) (Figure 2a,c). However, when the male and female mice were analyzed separately, only the female mice exhibit statistically significantly differences. The levels of PSD95 also show a possible trend of been partially restored by ANKHD1 (*p* = 0.058) (Figure 2a,d), however such trend was not evident when the male and female mice were analyzed separately. We did not detect effects of ANKHD1 on the levels of phosphor-CREB (P-CREB) when compared the Tau^PS19^+*ANKHD1* mice to the control or the Tau^PS19^ mice (Figure A3), although a separate study had previously reported a decrease in P-CREB levels in the hippocampal region in PS19 mice [35].

**Figure 3:**
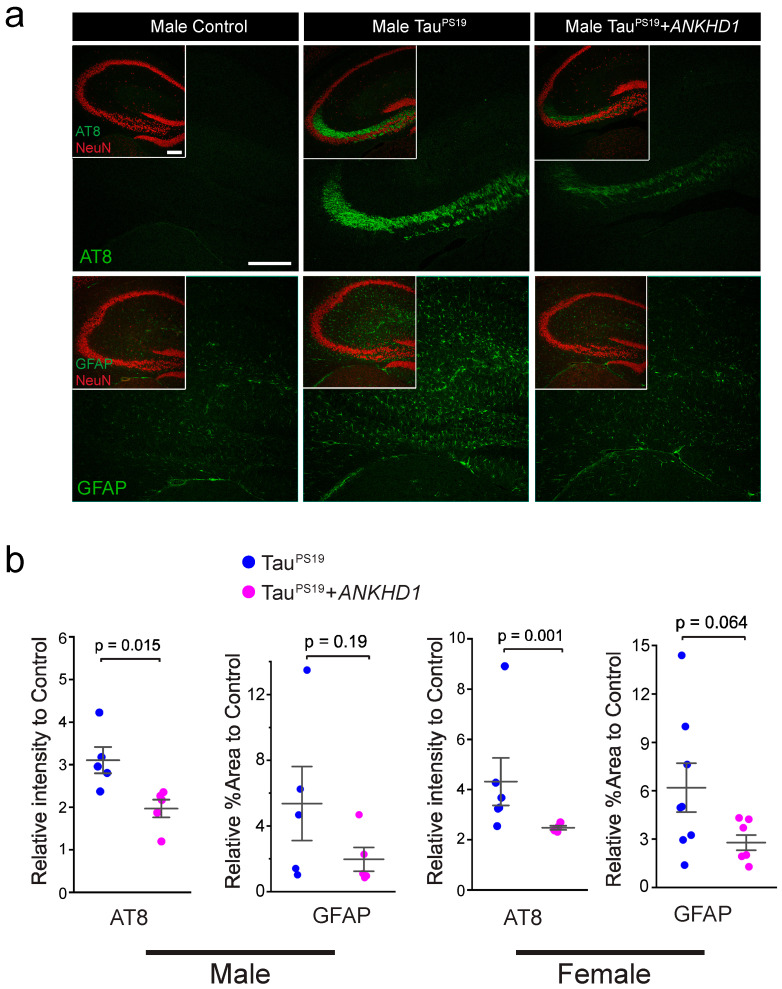
ANKHD1 co-expression reduces phosphorylated Tau in the brain of TauP301S transgenic mice at 6 months of age. (**a**) Representative confocal images of mouse brain hippocampus regions of Control, Tau^PS19^, and Tau^PS19^*+ANKHD1* mice. Mouse brain sections were stained with AT8 (anti-Tau-phospho-Ser202 and phosho-Thr205), anti-GFAP, and rabbit anti-NeuN antibodies, respectively, to detect phosphorylated Tau, gliosis, and nuclei of mature neurons. Scale bar, 40 µm. (**b**) AT8 and GFAP (n = 5 for both male and female of all genotypes) intensities in the brain sections from both male and female mice were quantified. Average fluorescence intensity of AT8 or anti-GFAP was normalized to their control littermates (CamK2a-Cre/+). The data was presented as the relative intensity ratio to the control mice.

The initial characterization of the TauPS19 mice has demonstrated that Tau-linked aggregates and related cellular deficits manifested as early as 3-month-old age and became prominent at 6-month-old age, although neuronal loss in hippocampus and cognitive decline were not evident at these ages [32]. We then examined the levels of Tau phosphorylation and gliosis in the hippocampal region in the mice brains at 6-month age. As expected, we observed prominent levels of phosphorylated Tau in the hippocampal CA3 region of the brain slices from the PS19 mice (pnp-Tau-P301S/+; CamK2a-Cre/+) marked by the reactivity to the AT8 antibody that specifically recognizes phosphorylated Tau proteins. Interestingly, in the brain sections from Tau^PS19^+*ANKHD1* mice, we observed a significant reduction in phosphorylated Tau levels in the same brain region of male (*p* = 0.01) and female (*p* = 0.001) mice (Figure 3).

Gliosis was a concomitant histopathological sign to neuronal loss in tauopathies [36]. In Tau^PS19^ transgenic mice, a mild increase in glial fibrillary acidic protein (GFAP)—a marker of astrocytes—can be detected throughout the brain as early as 3 months of age, with a pronounced elevation observed by 6 months [32]. To assess whether ANKHD1 can suppress glial activation induced by mutant Tau, we examined percentage GFAP-positive area in the hippocampus. Compared to PS19 mice, TauPS19+*ANKHD1* mice exhibited a trend toward reduced GFAP-positive area, though the difference did not reach statistical significance (*p* = 0.19 in males and *p* = 0.064 in females) (Figure 3).

**Figure 4:**
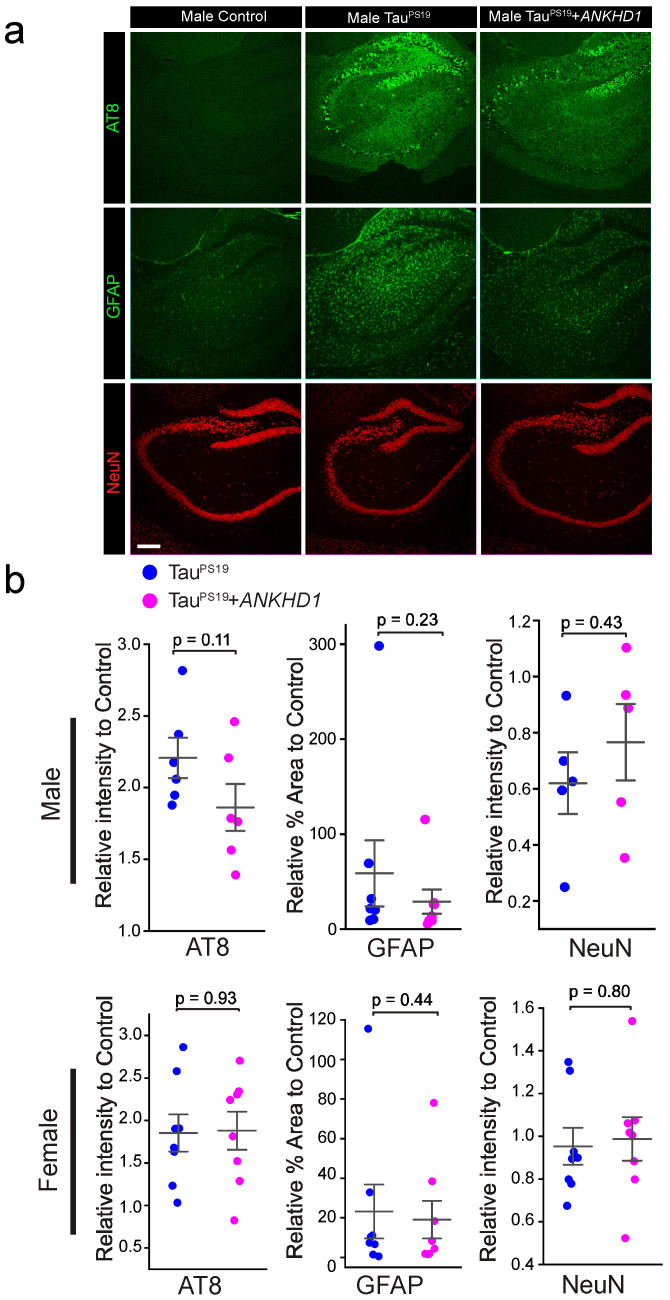
The effect of ANKHD1 co-expression on Tau phosphorylation and gliosis in TauP301S transgenic mice at 9 months of age. (**a**) Representative confocal images of mouse brain hippocampus of Control, Tau^PS19^, and Tau^PS19^+*ANKHD1* mice. Mouse brain sections were stained with AT8 (anti-Tau-phospho-Ser202 and phosho-Thr205), anti-GFAP, and mouse anti-NeuN antibodies, respectively, to detect phosphorylated Tau, gliosis, and nuclei of mature neurons. Scale bar, 40 µm. (**b**) AT8, GFAP and NeuN (n= 6 for male and n = 8 for female) intensities in the brain sections were quantified. Average fluorescence intensities of NeuN, AT8, or anti-GFAP were normalized to their control littermates. The data were presented as the relative intensity ratio to the control mice.

We extended the histological analysis to 9-month-old mice using the same assays as in the 6-month-old cohort; no statistically significant differences were observed between TauPS19+*ANKHD1* and TauPS19 mice, except for a minor trend of reduction in phosphorylated Tau in TauPS19+*ANKHD1* male mice (*p* = 0.11) (Figure 4).

### 2.3. ANKHD1 Partially Restores Cognitive Functions in the PS19 Mice

**Figure 5:**
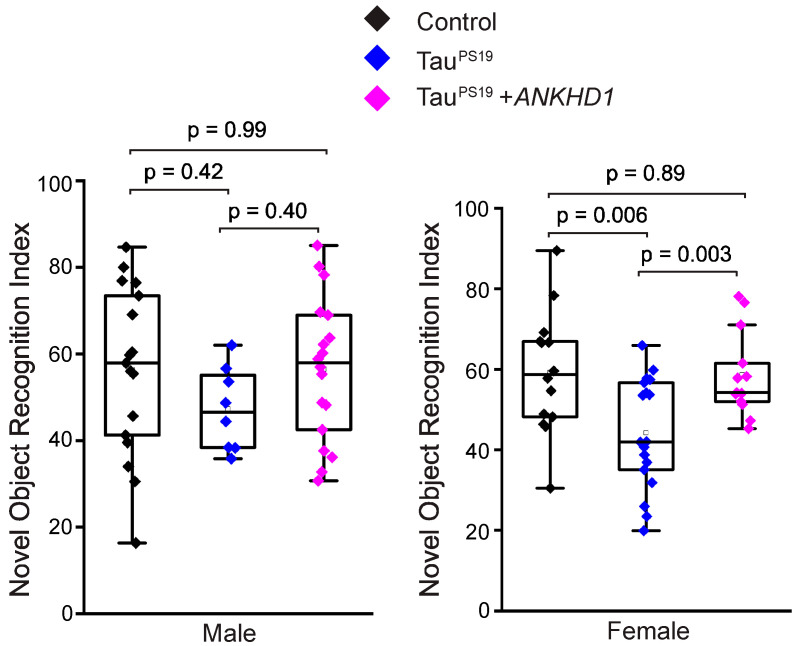
ANKHD1 co-expression rescues the defective NOR in TauP301S transgenic female mice at 9-month of age. Control, Tau^PS19^, and Tau^PS19^+*ANKHD1* mice were tested for their ability to recognize novel objects following standard protocol. Novel Object Recognition Index from the male and the female cohorts were presented (Male mice, n = 17 for the control, n = 8 for the Tau^PS19^, and n = 18 for the Tau^PS19^+*ANKHD1* mice; female mice, n = 12 for the control, n = 16 for the Tau^PS19^, and n = 12 for the Tau^PS19^+*ANKHD1* mice).

Tau pathology in the brain leads to functional impairments, and previous studies have shown that Tau^PS19^ mice exhibit cognitive deficits by 9 months of age, as assessed by the novel object recognition (NOR) [37] and Y-Maze [38] behavioral tests. We next tested whether the suppression of neuropathology in the Tau^PS19^ mice by ANKHD1 at 6-month age may correlate to improved cognitive functions at 9-month age. TauPS19 female mice showed significant drop in NOR index compared to control mice (44 vs. 59). We found that the NOR index has increased from 44 in the Tau^PS19^ mice to 58.5 in the TauPS19+*ANKHD1* mice (Figure 5). However, the reduction in NOR in the Tau^PS19^ male mice was not significant. Nevertheless, TauPS19+*ANKHD1* male mice showed a NOR index indistinguishable to the control mice (Figure 5). In our Y-Maze tests, neither male nor female 9-month-old Tau^PS19^ mice showed detectable deficit, preventing us from assessing potential rescue effects of ANKHD1 in this behavioral paradigm (Figure A5).

## 3. Discussion

Our previous work demonstrated that overexpression of Mask, the *Drosophila* homolog of mammalian ANKHD1 and ANKRD17, protects against neurodegeneration caused by human Tau and FUS in the fly eye model [18]. Mechanistically, *Mask* was found to enhance autophagic flux, which was essential for its neuroprotective effects. Given the strong sequence conservation between *Mask* and ANKHD1—particularly within their ankyrin repeat and KH domains—we hypothesized that ANKHD1 may play a similar protective role in mammalian systems.

In this study, we provide evidence that ANKHD1 promotes autophagy in cultured cells and modestly alleviates neuropathology in a mouse model of Alzheimer’s disease. In HEK293 cells, ANKHD1 overexpression reduced p62 levels, mirroring the effects of *Mask* upregulation in *Drosophila* larval muscle. However, in the hippocampus of transgenic mice—where ANKHD1 was only moderately overexpressed (Figure 2)—we did not observe consistent reductions in p62. While p62 is commonly used as a readout of autophagic activity, it is an indirect and context-dependent marker influenced by transcriptional regulation, proteasomal degradation, and general proteostasis. Therefore, p62 levels alone are insufficient to definitively assess autophagic flux. Future studies using complementary approaches such as LC3-II quantification and in vivo autophagy reporter assays will be critical to clarify the mechanistic role of ANKHD1 in modulating autophagy in the mammalian brain.

**Figure A2:**
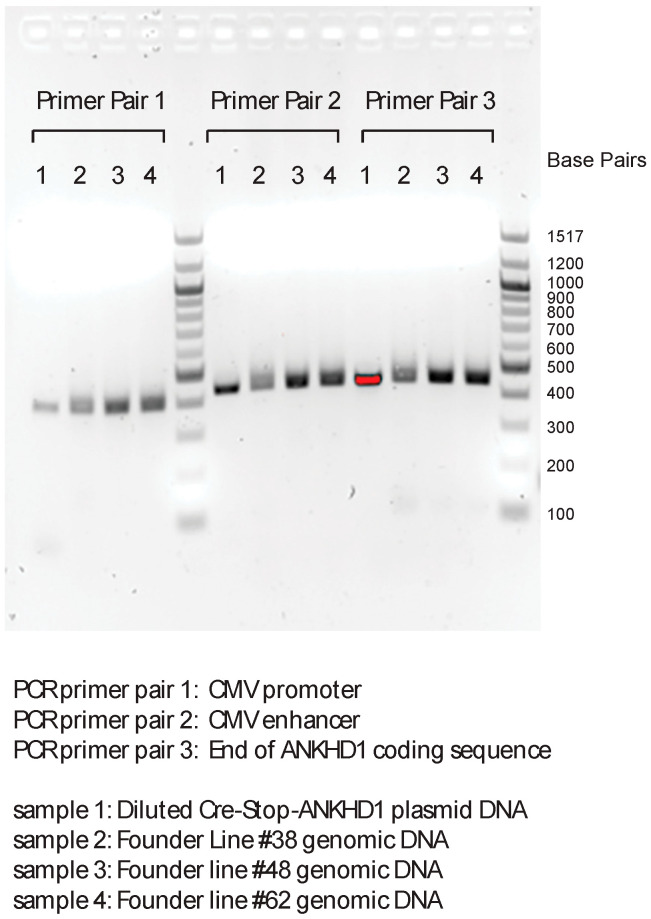
Validation of the sequence integrity of the Cre-Stop-*ANKHD1* transgene insertion. Diluted DNAs of Cre-Stop-ANKHD1 plasmid or genomic DNAs from three independent transgenic *Cre-Stop-ANKHD1* founder lines were subjected to PCR reactions with three primer pairs. The three primer pairs encompass the promoter region to the 3′ end of the ANKHD1 cDNA sequence in the *Cre-Stop-ANKHD1* transgene, as listed in the figure.

**Figure A3:**
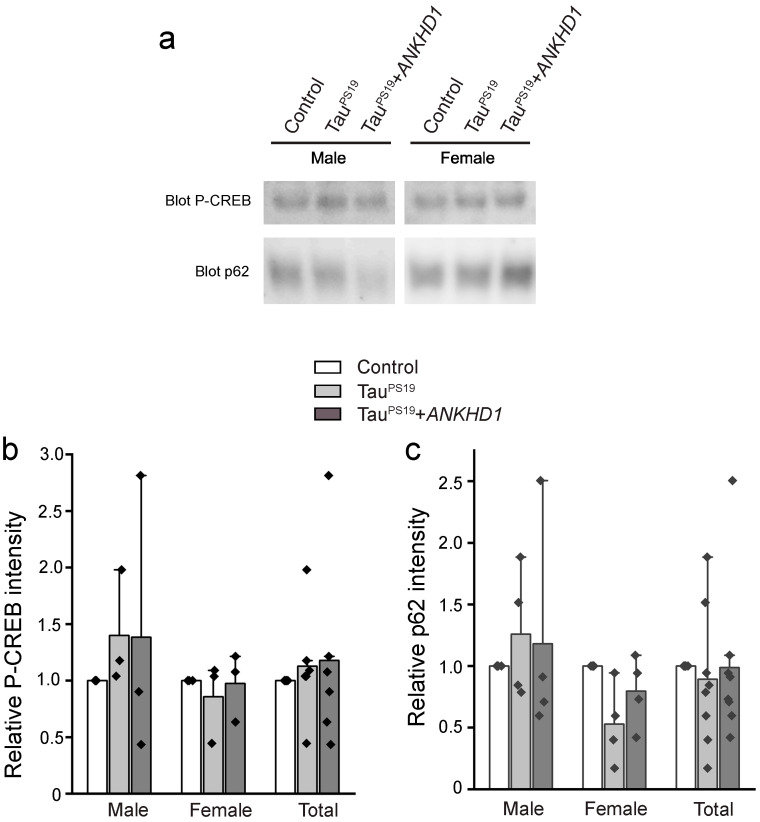
P-CREB and p62 abundance in the brains of control, TauP301S, and TauP301S + *ANKHD1* mice. (**a**) Representative Western Blot images of brain hippocampal lysate from the Control, Tau^PS19^, and Tau^PS19^+*ANKHD1* mice. Anti-p62 and anti-P-CREB antibodies were used to detect p62 and P-CREB protein. The same amount of total protein was loaded to each lane. (**b**) Quantification of P-CREB intensity on the immunoblot (n = 3 for both male and female mice). (**c**) Quantification of p62 intensity on the immunoblot (n = 4 for both male and female mice). The same amount of total protein was loaded to each lane.

Despite the structural homology between ANKHD1 and *Mask*, the neuroprotective effects of ANKHD1 in mice were modest compared to the robust outcomes observed in *Drosophila* models. This discrepancy is unlikely to be due to issues with transgene integrity, as sequencing confirmed the correct sequence from promoter to 3′ end in the transgenic constructs (Figure A2). We propose that the limited efficacy may be attributed to relatively low expression levels of ANKHD1 in the mouse hippocampus, particularly when compared to the strong overexpression achieved in flies or cultured cells. In our mouse model, ANKHD1 expression increased by approximately 40% in females (Figure 2), which may be insufficient to elicit the full protective potential. Thus, future strategies aimed at enhancing ANKHD1 expression—either globally or in targeted brain regions—may be necessary to fully assess its therapeutic capacity in the context of tauopathy.

**Figure A4:**
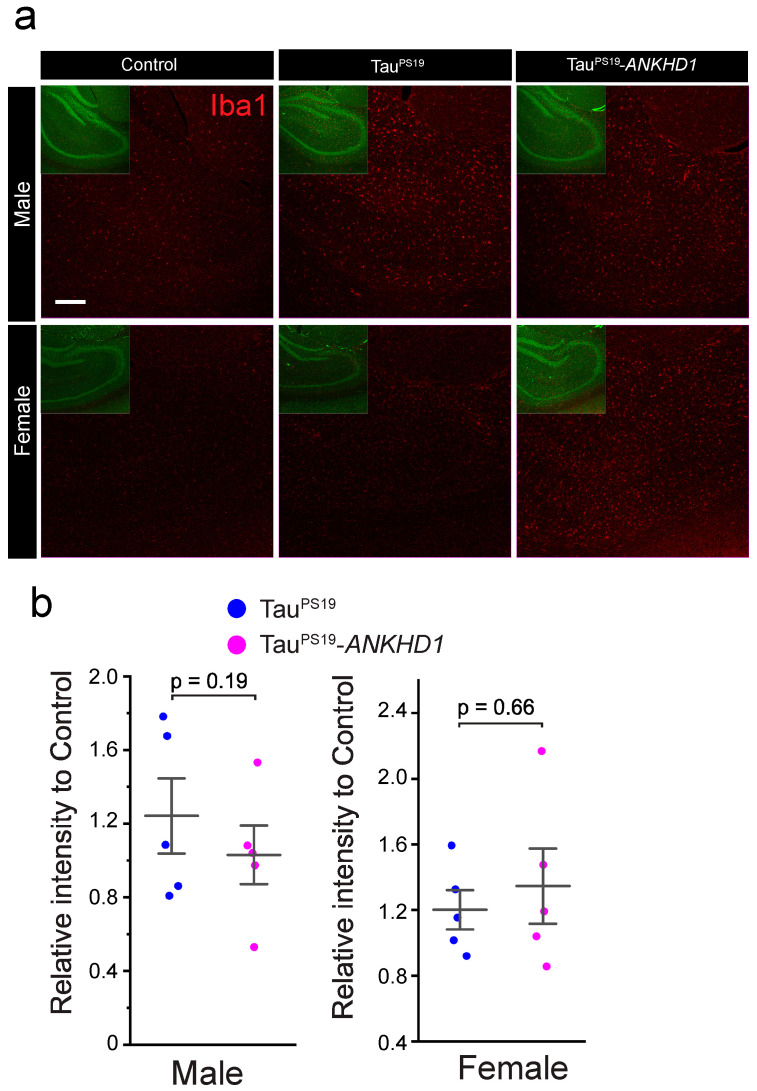
The effect of ANKHD1 co-expression on Iba1 abundance in brain ofTauP301S transgenic mice. (**a**) Representative confocal images of mouse brain hippocampus of Control, Tau^PS19^, and Tau^PS19^+*ANKHD1* mice. Mouse brain sections were stained with anti-Iba1 antibody (Cell Signaling) to detect microglia in the brain. Scale bar, 40 µm. (**b**) Iba1 intensity in the brain sections from male and female mice were quantified. The average fluorescence intensity of Iba1 was normalized to their control littermates. The data were presented as the relative intensity ratio to the control mice.

**Figure A5:**
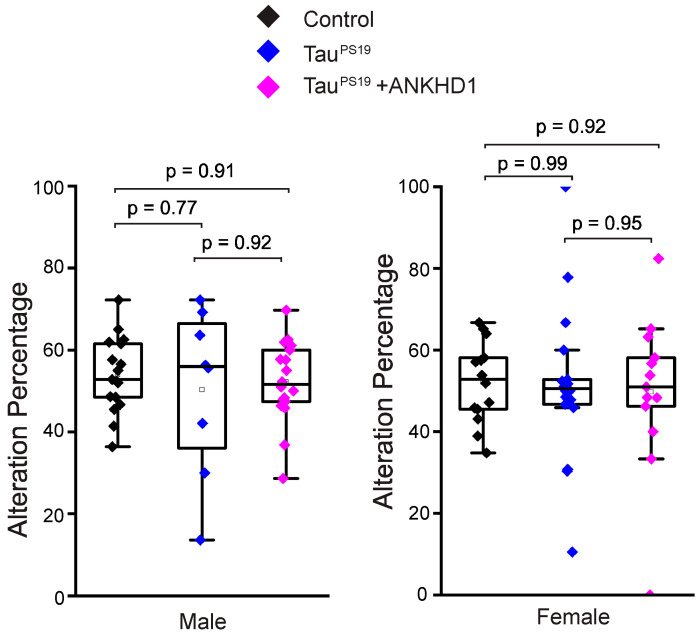
Y-Maze test of 9-month-old mice. Control, Tau^PS19^, and Tau^PS19^+*ANKHD1* mice were subjected to Y-Maze test following standard protocol. Alteration percentage from the male and the female cohorts were presented.

Interestingly, our findings also highlight potential sex-specific effects of ANKHD1. At 6 months of age, only female PS19+ANKHD1 mice exhibited a statistically significant increase in ANKHD1 expression, accompanied by a modest elevation in synaptophysin levels (Figure 2). Both male and female PS19+ANKHD1 mice showed reduced Tau hyperphosphorylation and gliosis at this age, but these effects were not sustained at 9 months, when no significant differences were observed in AT8, GFAP, Iba1, or NeuN levels between PS19 and PS19+ANKHD1 mice (Figure 4 and Figure A4). These findings raise important questions about the temporal dynamics of ANKHD1-mediated neuroprotection, suggesting that its efficacy may be strongest during early or mid-stage pathology. Future studies using time-course analysis and inducible expression systems will help define the critical window during which ANKHD1 exerts its protective effects.

Sex differences in Alzheimer’s disease (AD) are well documented, with women exhibiting a higher lifetime risk than men. Increasing evidence indicates that the loss of ovarian hormones during perimenopause contributes to this heightened vulnerability [39]. In cellular and animal models, estrogen has been shown to exert protective effects against AD-related pathology [40,41,42]. In addition to hormonal influences, sex differences in gene expression, alternative splicing, and autophagy [43]—affecting both sex chromosomes and autosomes—may underlie distinct protective or detrimental mechanisms in neurodegeneration. Epigenetic mechanisms, including the action of estrogen [44], non-coding RNAs, and microRNAs, further shape sex-specific gene expression profiles across the genome. Thus, sex differences in neurodegenerative diseases may reflect a complex interplay between hormonal status and differential gene regulation involving both autosomes and sex chromosomes [39].

Our findings further support a sexually dimorphic response in PS19 tauopathy mice. Previous studies reported that male PS19 mice exhibit more severe neuropathological features—such as accelerated weight loss, increased AT8 immunoreactivity, and heightened astrocyte activation—compared to females [45]. Yet, male PS19 mice often fail to show cognitive impairments in the Morris Water Maze (MWM), while female mice display significant deficits by 12 months of age [45]. In our study, using the Novel Object Recognition (NOR) test, we observed that female PS19 mice exhibited cognitive impairments as early as 9 months, whereas male PS19 mice performed comparably to wild-type controls. These findings are consistent with earlier reports and underscore the importance of analyzing behavioral data in a sex-specific manner. Notably, a previous study by Zampar et al. [46] did not detect NOR deficits in PS19 mice, likely due to combined analysis of males and females, which may have masked meaningful sex-specific differences.

Importantly, co-expression of ANKHD1 in female PS19 mice led to a statistically significant improvement in cognitive performance, as measured by the NOR test. In contrast, the absence of cognitive impairment in male PS19 mice at this age precluded evaluation of ANKHD1’s protective effects in males. Nevertheless, the observed improvement in females correlated with increased ANKHD1 expression at earlier time points, suggesting a potential link between ANKHD1 upregulation and cognitive resilience in female mice.

Finally, while our current data suggest that ANKHD1-coexoression offers modest neuroprotection in the context of Tau pathology, it remains to be determined whether ANKHD1 overexpression has broader functional benefits. In *Drosophila*, Mask regulates microtubule stability [47], thereby influencing synaptic morphology and function, and potentially supporting axonal transport—an essential mechanism for mitigating Tau toxicity [32]. Overexpression of Mask in a subset of dopaminergic neurons significantly extended both health span and lifespan in flies [48]. Whether ANKHD1 exerts similar gain-of-function effects in mammalian neurons is not yet clear. Preliminary observations indicate that neuronal expression of ANKHD1 alone does not cause overt phenotypic abnormalities in mice. However, a more comprehensive analysis—particularly with long-term or cell-type-specific overexpression—will be necessary to determine whether ANKHD1 influences selective autophagy, synaptic integrity, homeostatic regulation, or longevity in both normal and disease contexts.

## 4. Materials and Methods

### 4.1. Generation of Cre-Inducible ANKHD1 Transgenic Mouse

To generate the Cre STOPGFP-ANKHD1-IRES-mCherry DNA construct, we first PCR the IRES sequence from the pCall-IRES-GFP plasmid (a gift from Dr. Hang Shi) and cloned this sequence into the Cre Stoplight 2.4 plasmid (Addgene item 37402, Watertown, MA, USA) in between the LoxP-GFP-STOP cassette and the coding sequence for mCherry. The resulting plasmid was then used as the vector to receive the insert of the ANKHD1 coding sequence that was cut out from the Mask1 plasmid (a gift from Dr. Barry Thompson). The final DNA construct was sent to Texas A&M Institute for Genomic Medicine for perinuclear injection. C57/B6 strain was used for the injection for generating the founder lines. ANKHD1 internal sequence at the 3′ end of the coding sequence was used for PCR genotyping (Forward primer: GCACGTGGGCACCTCATATT; and Reverse primer: GGTACCTTCTGGGCATCCTT), Myogenin locus was used as the control for genotyping (Forward primer: TTACGTCCATCGTGGACAGC; and Reverse primer: TGGGCTGGGTGTTAGCCTTA). Other primers used for confirming the integration of the transgene are: TransgeneCMVFor: CCATGGTGATGCGGTTTTGGCA; TransgeneGFPRev: CCTCCATGCGGTACTTCATGGT; TransEnhancerF1: GCCAGATATACGCGTTGACATT; TrsnsEnhancerR1: GCTATCCACGCCCATTGATGTA.

### 4.2. Cell Culture and Transfection

HEK293 cells were cultured in 1× DMEM (Gibco 11966-092, Grand Island, NY, USA) supplemented with 10% FBS, 1× NEAA, 110 mg/L Sodium Pyruvate, and 100 U/mL Pen-Strep antibiotics mix under optimal conditions (37 °C, 5% CO_2_). Cells were transfected at an 80% confluency with CreSTOPGFP-ANKHD1-IRES-mCherry plasmid alone or together with CMV-Cre plasmid (Addgene 37402) using Lipofectamine3000 transfection reagent (Thermo Fisher L3000015, Waltham, MA, USA) following the protocols recommended by the manufacturer.

### 4.3. Western Blot Analysis

Transfected HEK293 cells cultured in a 12-well plate were harvested 48 h after transfection. The cells were quickly rinsed with 1× BS first and then incubated with SDS-Urea lysis buffer (9:2:1 of 100 mM NaCl, 1 mM EDTA, 100 mM Tris-HCl pH 8.0 Buffer, 10% SDS and 8 M Urea) in the wells (400 µL lysis buffer/well for a 12-well plate). The lysate was then treated with DTT (0.1 M), mixed with 4× SDS loading buffer, boiled for 5 min and centrifuged at 15,000× *g* for 5 min. The supernatant containing proteins was loaded and resolved on a 4–20% gradient gel (Bio-Rad 17000436, Hercules, CA, USA). Hippocampus was dissected out of the left or the right hemisphere of the mouse brain, snap frozen in liquid nitrogen and kept at −80 °C for storage before Western blot analysis. The hippocampal tissue was lysed in SDS-Urea lysis buffer (9:2:1 of 100 mM NaCl, 1 mM EDTA, 100 mM Tris-HCl pH8.0 Buffer, 10% SDS and 8 M Urea). Protein concentrations were measured using the Bio-Rad DC Protein Assay Kit (500011) on a SpectraMax M5 Microplate Reader (Molecular Devices, San Jose, CA, USA). Samples were then normalized to equal protein concentrations, treated with 0.1 M DTT, mixed with 4× SDS loading buffer, boiled for 5 min, and centrifuged at 15,000× *g* for 5 min. Equal volumes—corresponding to equal total protein amounts—were then loaded onto SDS-PAGE gels.

The following primary antibodies were used for immunoblotting: rabbit anti-ANKHD1 (1:1000, Biorbyte orb374007, Claremont, CA, USA), rabbit anti-p62 (1:1000, a gift from Dr. Sheng Zhang), mouse anti-GAPDH (1:10,000, Abcam Ab181602, Waltham, MA, USA), rabbit anti-PSD95 (1:1000, Cell Signaling 3450, Danvers, MA, USA), rabbit anti-synaptophysin (1:1000, Cell Signaling 36406) and rabbit anti-phosphor-CREB (1:1000, Cell Signaling 9198). All HRP-conjugated secondary antibodies (from Jackson ImmunoResearch, West Grove, PA, USA) were used at 1:5000 dilution. Data was collected using a ChemiDoc MP Image System (Bio-Rad) and quantified using ImageQuant TL 7.0 (GE, Cincinnati, OH, USA).

### 4.4. Immunohistochemistry

Mice brains were dissected at appropriate ages, fixed and embedded as previously described [49]. All mice were deeply anesthetized with 5% isoflurane, and the whole brains were dissected, rinsed with cold 1× PBS and then fixed in 4 mL of 4%PFA in 1× PBS at 4 °C O/N and then incubated in 10 mL of 30% sucrose in 1× PBS at 4 °C till the brains sink to the bottoms of the tube. The fixed brains were dried with Kim Wipe tissues and imbedded in OCT, freeze and stored at −80 °C till they were sectioned.

The brain blocks were moved to −20 °C for one hour before sectioning. 20 μm sections encompassing the hippocampus were collected into each well of 12-well plates filled with 1× PBS buffer. The free-floating sections were used for immunohistochemistry. Two consecutive sections were kept in a single well and washed with 1× PBS 3 times, followed by 0.1% Triton X-100/1× PBS (wash buffer) 3 times, and then incubated with primary antibodies that were diluted in the blocking buffer (1× PBS containing 0.1% Triton X-100, 2% goat serum, and 2% BSA) at 4 °C O/N. The following day, sections were rinsed in wash buffer for 3 times and then incubated with fluorophore conjugated secondary antibodies for 2 h at room temperature followed by three washes in the wash buffer. The sections were placed on the slides, mounted in SlowFade Gold antifade reagent (Invitrogen S36936, Carlsbad, CA, USA) and covered with coverslips for confocal microscope observation. Primary antibodies used in this study were as follows: mouse anti-phosphos-Tau Ser-202, Thr205 (1:100, AT8-biotin, Thermo Scientific MN1020B), rabbit anti-GFAP (1:1000, Cell Signaling 3670), mouse anti-NeuN (1:500, Millipore Sigma MAB377, Burlington, MA, USA), rabbit anti-NeuN (1:1000, Cell Signaling 24307), and rabbit anti-Iba-1 (1:1000, Cell Signaling 17198). For secondary antibodies, 488-, Cy3- or Cy5-conjugated donkey anti-species antibodies were used (all 1:1000, Jackson ImmunoResearch).

### 4.5. Confocal Microscopy and Quantification

Single-layer or z-stack confocal images were captured on a Nikon (Tokyo, Japan) C1 confocal microscope. Images shown in the same figure were acquired using the same gain from samples that had been simultaneously sectioned and stained. The mean intensities of phosphorylated Tau (AT8), NeuN, and Iba1 were measured using NIS-Elements imaging software 3.2 (Nikon, Melville, NY, USA). GFAP-positive area encompassing the hippocampus was measured in FIJI (ImageJ, version 1.54p) by applying a consistent threshold across each littermate group (control, Tau^PS19^, and Tau^PS19^+*ANKHD1*) to minimize subjectivity. The sectioning, immunostaining and image acquisition were performed in mice grouped based on their genotypes- littermates (or with close date of birth) of control, Tau^PS19^ and Tau^PS19^+*ANKHD1*. Within each group, the signal intensity of the Tau^PS19^ and Tau^PS19^+*ANKHD1* samples were normalized to the control sample.

### 4.6. Y-Maze

This test is based on the intrinsic tendency of rodents to explore a new environment [50]. Normal rodents will prefer to experience a different arm of the maze than the one they visited on their previous entry. Following previously established protocols [49], the mice were tested in a three-arm maze containing 3 equal arms of 35 cm length, 7 cm width, and 15 cm height, attached at 120-degree angles. Each arm of the maze is labeled as arm A, B, or C. In each session, the animal is placed in the center and allowed to freely explore the three arms for 8 min. Number of arm entries and number of alternations are scored live in order to calculate the percent alternation. An entry is considered as occurred when all four limbs of the mouse are within the arm. The alternation percentage is calculated as [(number of spontaneous alternations)/(number of possible triads)] times 100. The maze is cleaned with Chlorhexidine solution between animals to eliminate odor traces. All videos were recorded with and analyzed by Any-maze Y-Maze protocol (Any-maze).

### 4.7. Novel Object Recognition

Novel Object Recognition (NOR) task was performed as described previously [49]. The method is based on the observation that rodents tend to spend more time exploring a novel object than one they have previously encountered. For object recognition, animals were first placed in the square chamber allowing free exploration for 10 min to habituate the environment. One hour later, the animals were put back in the chamber containing two identical objects, and the animal was given 5 min to explore. Another hour after this training, the animal was returned to the chamber, and this time, one object was replaced with a novel object that had not been previously encountered, and the animal was allowed to explore for 5 min. A video camera was used to record movement in the chamber, and the time spent exploring each object was scored by Any-Maze NOR. The time spent exploring the novel object relative to the familiar one served as a measure of memory (Novel Object Recognition Index). To eliminate any olfactory cues, the chamber and objects were thoroughly cleaned after each use.

### 4.8. Data and Statistical Analysis

As the mouse cohort aged, experiments were conducted in groups of three mice, each including one animal from each genotype—control, Tau^PS19^, and Tau^PS19^+*ANKHD1*—using littermates or mice with closely matched birth dates. At either 6 or 9 months of age, brain sectioning, antibody staining, and confocal imaging were performed concurrently for all mice within a group. To account for experimental variability, signal intensities from Tau^PS19^ and Tau^PS19^+*ANKHD1* samples were normalized to their respective control, with the control value set to 1. This normalization approach helped minimize variations arising from differences in experimental timing, antibody conditions, and procedural inconsistencies.

Statistical analysis was performed, and graphs were generated using OriginPro (OriginLab, Northampton, MA, USA). Each data set was tested for normal distribution and then compared with other samples in the group (more than two) using one-way ANOVA followed by post hoc analysis with Tukey’s test, or with the other sample in a group of two using t-test. Multiple comparisons were additionally analyzed using Bonferroni correction, which confirmed that the results obtained from Tukey’s tests did not yield false positive. All Bar Overlap and Scatter Interval plots show mean ± s.e.m with all data points indicated in each graph.

## Data Availability

The original data supporting the reported results were submitted with the initial manuscript and are available upon request from the corresponding authors.

## Abbreviations

The following abbreviations are used in this manuscript:

MT	microtubule
PHF	paired helical filaments
PS19	Tau^PS19^, or pnp-Tau-P301S
NOR	Novel Object Recognition
GFAP	glial fibrillary acidic protein (GFAP)
AT8	phosphorylated Tau
